# Reducing the diagnostic and treatment gap for priority mental, neurological and substance misuse disorders in primary care in rural Kenya: Results of a stepped wedge cluster randomized trial from the EPINA study

**DOI:** 10.1371/journal.pone.0352643

**Published:** 2026-08-17

**Authors:** Mary A. Bitta, Symon M. Kariuki, Daniel M. Mwanga, Mercy Atieno, Nuru Kibirige, Elias Fondo, Gergana Manolova, Neerja Chowdhary, Tarun Dua, Belinda Lennox, Charles RJC Newton, on behalf of the EPInA Study Group

**Affiliations:** 1 Clinical Research-Neurosciences, KEMRI/Wellcome Trust Research Programme, Centre for Geographic Medicine Research (Coast), Kilifi, Kenya; 2 Department of Psychiatry, University of Oxford, Oxford, United Kingdom; 3 Brain and Mind Institute, Aga Khan University, Nairobi, Kenya; 4 Department of Public Health, Pwani University, Kilifi, Kenya; 5 Africa Population Health Research Center, Nairobi, Kenya; 6 Department of Mathematics, University of Nairobi, Nairobi, Kenya; 7 Department of Health, County Government of Kilifi, Kilifi, Kenya; 8 Department of Mental Health and Substance Use, World Health Organization, Geneva, Switzerland; Ladoke Akintola University of Technology Teaching Hospital: LAUTECH Teaching Hospital, NIGERIA

## Abstract

In resource-limited settings, improving care for mental, neurological and substance misuse (MNS) disorders is essential. This study evaluated the feasibility and effectiveness of implementing all adult modules of the WHO mental health Gap Action Programme Intervention Guide (mhGAP-IG) in primary healthcare (PHC) in rural Kenya through training non-specialist primary healthcare workers. We conducted a stepped-wedge cluster randomized trial across 127 public primary care facilities in Kilifi County, Kenya. Facilities were grouped into 16 clusters and randomly assigned to one of 16 monthly intervention start dates between August 2021 and November 2022. All clusters began in the control condition and sequentially crossed over to the intervention. The intervention comprised a 10-day mhGAP-IG training followed by quarterly supportive supervision. The primary outcome was the monthly rate of clinical consultations for MNS disorders per 100,000 outpatient consultations. Secondary outcomes included healthcare worker stigma and patient-level measures of illness severity, disability, and quality of life. A total of 172 healthcare workers were trained, and 5,320,423 adult outpatient consultations were recorded. Compared to the control period, the monthly rate of epilepsy diagnoses increased from 191.35 to 395.49 per 100,000 consultations (crude absolute increase 204.13 per 100,000), with an adjusted intervention effect of β = 119.19 (95% CI 34.70–203.67; p = 0.006). Depression diagnoses increased from 1.41 to 15.24 per 100,000 consultations (crude increase 13.82 per 100,000; β = 9.70, 95% CI 5.69–13.71; p < 0.001), dementia from 0.25 to 5.58 (crude increase 5.33 per 100,000; β = 4.11, 95% CI 2.51–5.71; p < 0.001), and substance misuse disorders from 0.04 to 2.14 (crude increase 2.10 per 100,000; β = 1.78, 95% CI 0.95–2.61; p < 0.001). Although crude increases were observed for psychosis (18.55 to 49.38 per 100,000), suicidality (0.17 to 0.46), and a decrease for other mental health complaints (108.44 to 81.15), the adjusted intervention effects for these outcomes were not statistically significant. Healthcare worker stigma and patient outcomes improved significantly. Large-scale implementation of mhGAP-IG in rural Kenyan PHC was feasible and associated with improved detection of MNS disorders and better provider and patient outcomes. Further studies in diverse settings are needed to assess generalizability.

**Trial registration:**

Pan African Clinical Trials registry [PACTR202011741472301]

## Background

Mental, neurological and substance misuse disorders (MNS) contribute substantially of the global burden of diseases, yet access to care remains limited in many low and middle-income countries (LMIC) where specialist services are scarce and largely concentrated in urban settings. As a result, a large proportion of individuals with MNS do not receive appropriate care, even when they present to primary care facilities, where healthcare workers often lack training and resources to assess and manage these conditions. These persistent treatment gap reflects broader health system constraints and highlights the need for scalable approaches to integrating mental healthcare into primary care [1].

In response, the World Health Organization (WHO) developed the mental health GAP Action Programme Intervention Guidelines (mhGAP-IG), a set of simplified, evidence-based clinical guidelines designed to support non-specialist healthcare workers in the assessment and management of priority MNS conditions [2]

Since its inception in 2008, the mhGAP programme has been used in over 100 countries for research, training, and service delivery [3,4]. Evidence from randomized and quasi-experimental studies shows that mhGAP-IG training improved provider knowledge, attitudes and confidence and may improve patient level outcomes. However, existing literature remains dominated by training-focused evaluations and studies targeting a limited number of disorders. Systematic reviews have highlighted that relatively few studies have evaluated mhGAP-IG implementation under routine service delivery conditions and there is limited evidence from large scale pragmatic trials assessing multiple mhGAP modules delivered concurrently in real-world primary care settings [5,6].

In Kenya, the mhGAP-IG has been contextualised and pilot tested in primary care settings. In Kilifi County in rural coastal Kenya, version 2 of the guidelines was adapted and pilot tested among primary healthcare providers, demonstrating improved knowledge after training [7]. In Makueni county, an adapted mhGAP-IG led to reduced disability, improved seizure control and created parental awareness of children’s mental health symptoms., narrowing the treatment gap [8]. These studies were however small in scale and addressed only selected modules, leaving a critical evidence gap regarding large-scale implementation of all modules in a real-world health system.

The present study addresses this evidence gap by using a stepped wedge cluster randomised design to evaluate the effectiveness of a large-scale implementation of the mhGAP-IG across all 156 public primary care facilities in Kilifi County, Kenya. Stepped wedge designs are particularly suited to evaluating interventions delivered at scale within routine health systems where phased implementation is required, as all clusters begin in the control condition and cross over to the intervention on a staggered schedule, meaning all sites ultimately receive the intervention [9] To our knowledge, this is the first stepped wedge cluster randomised trial to evaluate real-world, system wide implementation of all adult mhGAP-IG modules in sub-Saharan Africa.

The study is embedded within the Epilepsy Pathways Innovation in Africa study (EPInA). The EPInA study is a multisite study in Kenya, Ghana and Tanzania that aims to improve outcomes for people with epilepsy through reducing stigma, preventing risk factors for epilepsy, promoting accurate diagnosis at all levels of care and timely access to evidence based and high-quality care. One pathway to achieving these goals was through training of primary healthcare workers on all modules of the mhGAP-IG. The child and adolescent mental and behavioural disorders module is being implemented and evaluated in a separate ongoing study [10]. Additional details of the EPInA study can be found at Epilepsy Pathway Innovation in Africa—Nuffield Department of Clinical Neurosciences (ox.ac.uk).

## Methods

### Study design

This study employed a stepped wedge cluster randomized trial (SW-CRT) design to evaluate the implementation of the Mental Health Gap Action Programme Intervention Guide (mhGAP-IG) in Kilifi County, Kenya. Clusters were defined based on geographical proximity to minimize the risk of contamination due to healthcare worker movement between facilities. Each cluster comprised eight facilities, except for one cluster with seven facilities due to the closure of a facility during the planning stage. The intervention rollout occurred over 16 months, from August 2021 to November 2022, with clusters randomly assigned to one of 16 start dates, spaced one month apart.

### Study setting

The trial was conducted across 127 out of 156 public primary care facilities in Kilifi County. All clusters provided usual care during the baseline period from January 2019 to July 2021. The guide has been contextualized and validated for use in the study setting. The trial was approved by the following Research Ethics Committees; WHO Ethics Review Committee [ERC.0003627], Scientific and Ethics Review Unit in Kenya [KEMRI/SERU/CGMR-C/186/4060], National Commission for Science, Technology, and Innovation [NACOSTI/P/21/13297] and Oxford Tropical Research Ethics Committee protocol number 11–20.

### Participants

Primary Care Facilities: Eligible facilities were functional public primary care centres with at least one full-time primary healthcare provider. “Functional” was defined as government-owned facilities operational and providing daily healthcare services throughout the study duration.

Healthcare Providers: Inclusion included licensed full-time staff at participating facilities expected to remain in post throughout the study period. Exclusion criteria included prior specialized training in mental, neurological, or substance misuse disorders, plans for retirement, known transfers to other facilities during the study period and inability or unwillingness to provide written informed consent to participate in the research study.

Patients: Patients were eligible if they were 18 years or older, capable of providing written informed consent, and newly diagnosed and managed by a trained primary healthcare worker during the study.

### Randomization and allocation concealment

Clusters were grouped based on geographical proximity to minimize inter-site contamination. Stratification was based on geographical grouping of clusters. Clusters were randomly ordered using a computer-generated random sequence in Stata version 17 to determine their intervention start date, with one cluster assigned to each of the 16 monthly start dates spanning August 2021 to November 2022. Given the one-to-one correspondence between clusters and intervention dates, random allocation was implemented by assigning clusters to the 16 monthly start dates using a computer-generated sequence. The randomization sequence was generated using Stata version 17 (C) by an independent statistician at the KEMRI Wellcome Trust Research Programme (KWTRP), who was not involved in participant recruitment or trial implementation. This sequence assigned each cluster to one of 16 predetermined intervention start dates.

Allocation concealment was maintained by restricting access to the randomization schedule. The allocation list was securely stored and was not accessible to trial staff involved in recruitment or outcome assessment. Cluster allocation was only revealed to implementation teams shortly before the intervention commenced in each cluster to allow logistical preparation. Randomization refers to the assignment of clusters to intervention start dates whereas allocation concealment refers to preventing foreknowledge of these assignments prior to implementation.

### Blinding

Blinding of primary healthcare workers was not feasible due to the need for advanced scheduling of work duties. However, research staff who were responsible for participant enrolment remained blinded to cluster allocation, ensuring that patients were enrolled without knowledge of their group assignments.

Patient recruitment followed the stepped-wedge cluster randomized design. Participants were enrolled consecutively from primary health care facilities before the intervention (mhGAP training) was delivered at each site. Participants were recruited during the control phase and followed over time in line with stepped-wedge design principles. Patients were recruited from outpatient consultations across all participating clusters. Recruitment continued at each site until the overall sample size was reached; the number of patients per site varied depending on flow and eligibility. Participants were blinded to whether their clinician had received mhGAP training.

### Intervention

The mhGAP-IG intervention involved training at least one non-specialized primary healthcare worker from each facility. Training spanned 10 consecutive working days, approximately 6 hours per day, combining didactic lectures with role-plays using case scenarios. The initial training day included consent procedures, baseline assessments, and a review of data collection tools. Three master trainers, supervised by WHO team members, conducted the training. These trainers were clinical officers with tertiary-level education and a minimum of three years of clinical experience in Kenyan primary care settings. They completed a four-week training course covering mhGAP-IG content and training manual utilization.

Prior to the intervention, standard care varied across sites and was based on clinician judgment, guided in part by national resources such as the essential medicines list and clinical protocols. No formal mental health training had been provided prior to the intervention. During the control phase, providers delivered usual care without structured mhGAP-IG support.

Post-training, primary healthcare workers received semi-structured supportive supervision from master trainers and three mental health practitioners based at Kilifi County’s psychiatric outpatient units. Supervision included face-to-face meetings every three months to discuss challenging cases and on-demand telephone support.

### Outcomes

*Primary Outcome*: The main outcome was the monthly rate of of clinical consultations for selected priority mental, neurological, and substance use (MNS) disorders at primary care facilities, expressed per 100,000 outpatient consultations, with the total number of outpatient consultations as the denominator.

*Secondary Outcomes*: These included changes in healthcare workers’ stigma levels—assessed through knowledge, attitudes, and behaviour—and patient-reported measures of illness severity, disability, and quality of life.

### Data collection

Primary outcome data were extracted from routinely collected hospital records. Trained field workers gathered anonymized patient-level data on illness severity, quality of life, and disability at the facility level, uploading it daily to a secure database hosted by the KEMRI Wellcome Trust Research Programme.

Healthcare workers’ knowledge about mental illness was evaluated at baseline and post-training using multiple-choice questions from the mhGAP-IG curriculum and the Mental Awareness Knowledge Schedule (MAKS). Attitudes and intended behaviours were measured using the Community Attitudes Towards the Mentally Ill Scale (CAMI) and the Reported and Intended Behaviors Scale (RIBS), respectively. These tools have been adapted and validated for use in Kilifi.

Patients assessed by mhGAP-IG-trained clinicians had their illness severity and improvement evaluated using the Clinical Global Impression scale. Quality of life and disability were measured using the WHO Quality of Life-BREF and the 12-item WHO Disability Assessment Schedule 2.0 (WHODAS 12), administered by trained research assistants. Disability assessments occurred monthly, while quality of life was evaluated bi-weekly over a six-month follow-up period.

### Sample size calculation

The primary outcome’s sample size was determined using the Hussey and Hughes approach, considering an estimated effect size, a within-cluster correlation coefficient of 0.05, an alpha level of 0.05, and 80% power. This intra-cluster correlation co-efficient is consistent with empirical estimates from cluster randomized trials where median ICC values are typically around 0.01–0.04 and the value of approximately 0.05 have been reported as representative in methodological studies [11–13]The calculation was based on the primary outcome (facility-level rates of consultation for MNS disorders). Formal sensitivity analyses using alternative ICC values were not undertaken and this is acknowledged as a limitation.

The sample size and power calculations were designed to detect improvements in the facility-level rate of clinical consultations for mental, neurological, and substance misuse disorders (MNSDs) following the intervention. Assuming a prevalence of 10.8% of all MNSD, a previous situational analysis indicated a baseline consultation rate for identified MNS disorders of ~2.5% [14]. We aimed to detect a minimum absolute increase of 9%, corresponding to a post-intervention rate of 11.5%, with a power of 80%. These differences represent clinically meaningful improvements based on programmatic expectations. For the patient level analysis, to account for an estimated 20% attrition rate, 250 participants were required. Ultimately, 261 participants were analysed.

### Statistical analysis

Baseline characteristics of healthcare workers and patients were summarized using descriptive statistics. Primary and secondary outcomes were analysed using linear mixed effects models. For the primary outcome, the model included fixed effects for intervention status and time, and random intercepts for clusters and facilities nested within clusters to account for within-cluster and within-facility correlations inherent in the stepped wedge design. Time was included as a categorical fixed effect to adjust for secular trends. The intervention effect therefore represents the average difference in outcome between intervention and control periods after accounting for time and clustering. The analysis assumed that the outcome was approximately continuous and normally distributed with residual dependence accounted for by the random effects structure. Formal sensitivity analyses using alternative model specifications were not undertaken. Subgroup analyses explored potential variations in intervention effects across different healthcare worker cadres. The trial reported is reported in accordance with the CONSORT extension for stepped wedge cluster randomized trials [14]. [9,14]

### Ethics approval and consent to participate

The study was approved by the World Health Organization Ethics Review Committee (ERC.0003627), the Kenya Medical Research Institute Scientific and Ethics Review Unit (KEMRI/SERU/CGMR-C/186/4060), the National Commission for Science, Technology and Innovation (NACOSTI/P/21/13297), and the Oxford Tropical Research Ethics Committee (protocol number 11–20). Written informed consent was obtained from all participating healthcare workers and patients enrolled in the study. For participants unable to provide written consent, informed consent was obtained using a thumbprint in the presence of an independent witness, who provided written confirmation of the consent process, in accordance with approved ethical guidelines.

## Results

### Primary outcome

**Fig 1:**
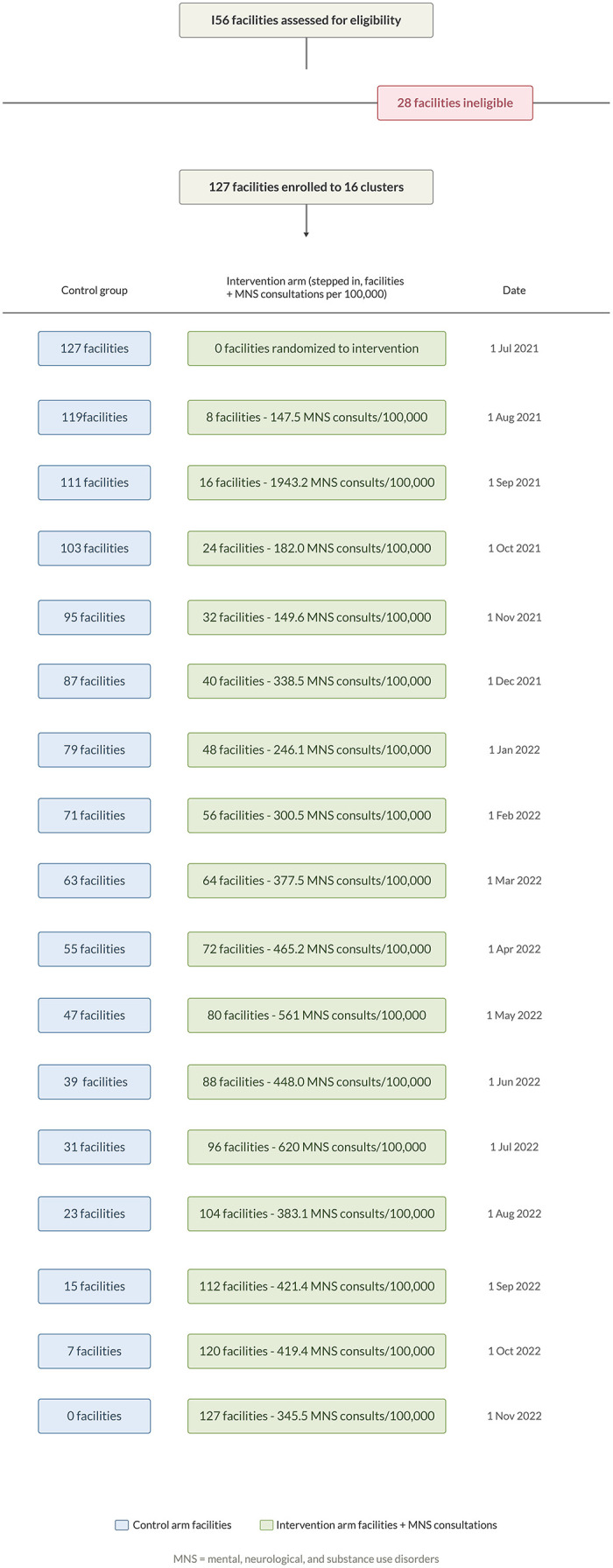
Flow diagram of study recruitment and cluster allocation. Flow diagram showing recruitment of primary healthcare facilities and their allocation across the 16 clusters in the stepped-wedge cluster randomized trial.

Between August 2021 and November 2022, a total of 172 primary healthcare workers from 127 primary care facilities were trained. During the study 5,320,423 adult outpatient consultations were recorded. A summary of the trial profile is provided in Fig 1.

The sociodemographic characteristics of the trainees are summarized in Table 1. Eight facilities were excluded in the final analysis for the following reasons: three facilities had a psychiatric referral facility within their units and it was difficult to reconcile the records of which patients were seen by the trainees and by the specialists based within these facilities, two facilities were based inside prisons and we could not collect follow-up data due to legal implications. The other three facilities could not provide follow up data as the physical records were destroyed through various causes such as vermin and floods.

**Table 1:** Characteristics of Primary Healthcare Workers who were trained on the mhGAP-IG Data are presented as n (%) for categorical variables and as mean (SD), median (IQR), and range for age. Percentages were calculated using the total baseline sample (N = 172), except where data were missing.

Characteristic	Category	n (%) or summary
Number of participants		172
Age (years)	Mean (SD)	39.7 (6.3)
	Median (IQR)	38.5 (35–43)
	Range	30–63
Sex	Female	102 (59.3)
	Male	70 (40.7)
Education level	Diploma	155 (90.1)
	Degree	9 (5.2)
	Certificate	8 (4.7)
Designation	Nurse	121 (70.4)
	Clinical Officer	51 (29.7)
Previous mhGAP training	No	171 (99.4)
	Yes	1 (0.6)

The total number of clinical consultations for adults (18 or older) at baseline for all facilities was 4,694,237 over 31 months of data collection, that is a mean of 1173.56 (SD = 1452.32) monthly clinical consultations per facility *vs* 626,186 over 16 months of intervention which was a monthly mean of 1144.77 (SD = 1075.49) per facility. There was no significant difference in the mean number of clinical consultations per facility between baseline and the intervention period (p = 0.65). The total number of diagnoses for all MNS at baseline was 13,318 (0.28%) and 2,412 (0.39%) post-intervention.

**Fig 2:**
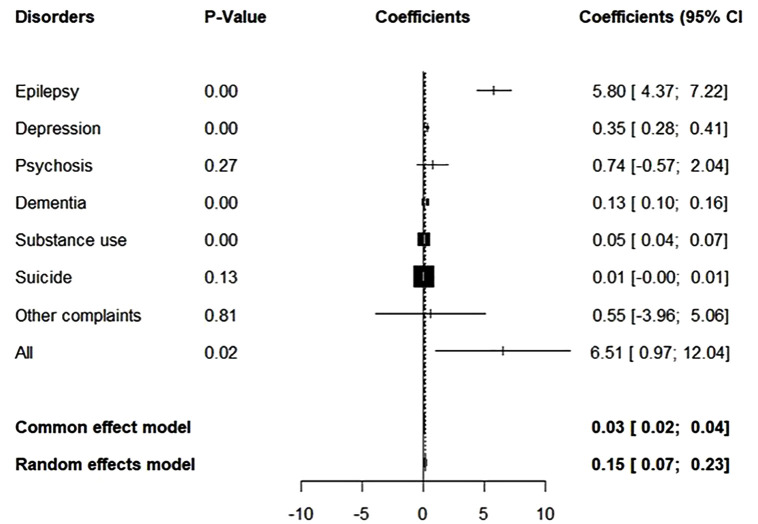
Adjusted intervention effects on consultation rates by disorder. Forest plot showing adjusted coefficients and 95% confidence intervals for the effect of mhGAP-IG implementation on monthly clinical consultation rates per 100,000 outpatient visits by disorder. The dashed vertical line indicates the null value.

Compared to baseline, the monthly rate of epilepsy diagnosis increased from 191.35 to 395.49 per 100,000 consultations (crude absolute increase of 204.13 per 100,000). The adjusted intervention effect from the linear mixed-effects model was β = 119.19 (95%CI = 34.70, 203.67 p = 0.006). For depression, the monthly rate of diagnosis increased from 1.41 to 15.24 per 100,000 consultations (crude absolute increase of 13.82 per 100,000). The adjusted effect estimate was β = 9.70(95% CI = 5.69, 13.71, p = 0.000). For dementia, the monthly rate of diagnosis increased from 0.25 to 5.58 per 100,000 consultations (absolute increase of 5.33 per 100,000 consultations). The adjusted estimate effect was β = 4.11(95% CI = 2,51, 5.71, p = 0.000) For substance misuse the monthly rate of substance misuse diagnoses increased from 0.04 to 2.14 per 100,000 consultations (crude absolute increase: 2.10 per 100,000). The adjusted estimate effect was β = 1,78(95% CI 0.95,2.61 p = 0.000). For psychosis, the monthly rate of psychosis diagnoses increased from 18.55 to 49.38 per 100,000 consultations (crude absolute change of 30.83 per 100,000), but the adjusted intervention effect was not statistically significant β = 24.72(95% CI = −50.68, 100.12 p = 0.520). For suicidality, the monthly rate of suicidality diagnoses increased from 0.17 to 0.46 per 100,000 consultations (crude absolute change: 0.29 per 100,000), though the adjusted intervention effect was not statistically significant β = −0.281(95% CI = −0.76, 0.20, p = 0.252). For other mental health complaints, the monthly rate of other significant mental health complaint diagnoses decreased from 108.44 to 81.15 per 100,000 consultations (crude absolute change: −27.30 per 100,000), but the adjusted intervention effect was not statistically significant β = −14.72 (95% CI = −276.95,247.51 p = 0.912) as summarized in Fig 2.

### Secondary outcomes

#### Trainee changes in levels of stigma.

*(i) Median change in knowledge about diagnosis and management of priority disorders as measured by mhGAP-IG multiple choice questions.*

**Fig 3:**
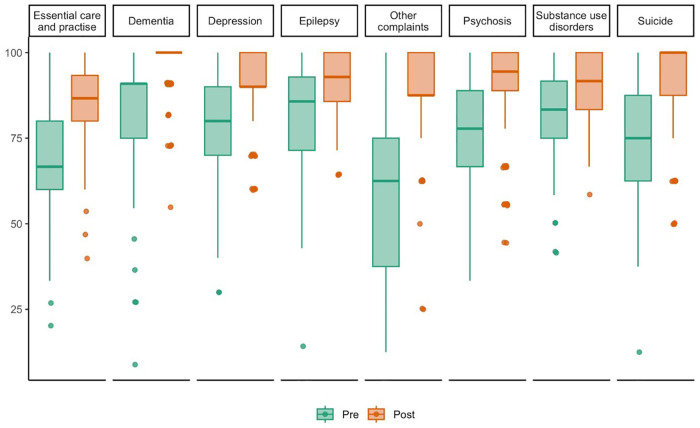
Healthcare worker knowledge scores before and after mhGAP-IG training. Box plots showing pre-training and post-training knowledge scores across eight clinical domains. Boxes show the interquartile range, the centre line indicates the median, whiskers indicate the range excluding outliers, and points represent individual observations.

The largest percentage point improvement of 31.3% was observed in the other significant mental health complaints module where the baseline median score was 62.5% (95% CI = 37.5–75) and the post intervention score was 93.8% (95% CI = 87.5–100). The percentage point improvements in the other modules were 25% for suicide, 20% for depression and essential care and practice modules, 16.6% in the psychosis module, 9.1% for dementia, 8.4% for substance misuse disorders and 6.9% for epilepsy as summarised in Fig 3. The baseline and post intervention scores and p-values for equality of medians test are provided in S1 Table.

*(ii) Changes in knowledge as measured by the Mental Awareness Knowledge Schedule*

In the multivariable models, general mental health-related knowledge β = 0.74 (95% CI = 0.52,0.96, p = 0.00) and knowledge about specific mental illnesses β = 0.18 (95%CI = −0.05,0.42, p = 0.00) improved post-intervention. This improvement in knowledge did not differ by age, sex, level of education or designation of the healthcare workers (S2 Table). Results of the univariable and multivariable analyses are provided in S2 Table.

*(iii) Changes in intended behaviour*

**Fig 4:**
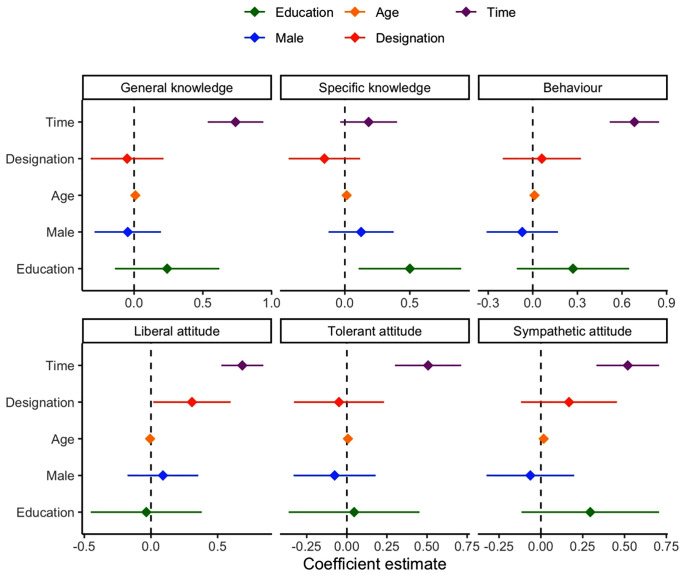
Associations between healthcare worker characteristics and stigma-related outcomes. Forest plots showing adjusted coefficients and 95% confidence intervals for the associations between healthcare worker characteristics and six stigma-related outcomes. The dashed vertical line indicates the null value.

Compared to baseline, primary healthcare workers reported significantly more positive intended behaviours towards people with MNS after the training β = 0.68 (95%CI = 0.48,0.88, p = 0.000) (Fig 4).

Positive behaviours included willingness to live nearby, work with and continue a relationship with a friend who developed mental health problems. Compared to healthcare workers with certificate level training (two years of formal training), the scores for positive intended behaviour were significantly higher for healthcare workers with diploma (3 years of training) or degree training (4 years of training) β = 0.26 (95% CI = 0.02,0.49, p = 0.03). There were no significant differences in the scores by age, sex or designation (S3 Table). Results for all univariable and multivariable analyses are provided in S3 Table.

*(iv) Changes in Attitudes*

In the multivariable models, healthcare workers were significantly more sympathetic β = 0.52 (95%CI = 0.30,0.75, p = 0.00) towards people with MNS post training (Fig 4). They were also significantly more tolerant β = 0.51 (95%CI = 0.28, 0.73, p = 0.00) and more liberal β = 0.26 (95%CI = 0.46, 0.89, p = 0.00) that is they were less likely to endorse items that involved the use of force or threats to handle patients. Compared to nurses, clinical officers were significantly more liberal post training β = 0.34, (95%CI = 0.10,0.58, p = 0.01). There were no correlations between the scores in the sympathetic and tolerant domains and any sociodemographic variables. Results for all univariable and multivariable analysis are provided in S4 Table.

### Patient level outcomes

#### Sociodemographic characteristics of patients at baseline.

A total of 261 patients were recruited in the study of whom 160 (61.3%) were female. Based on the calculated required sample size, we recruited 11 extra patients because recruitments were done simultaneously in multiple facilities and reconciliations were done at the end of each day, therefore on the last day, more patients than necessary were recruited. The mean age was 36.68 years (SD = 14.04). Of those who were recruited, 198 (75.86%) were self-referrals, 27 (10.34%) were referred by community health volunteers, 34 (13.03%) were referred by traditional health practitioners and there were no data on the point of referral for 2 (0.77%) patients. Two hundred and forty-two (92.72%) were treated at the primary care facilities without needing further referrals, 15 (5.75%) were referred to tertiary care and 4 (1.53%) were treated then referred to tertiary facilities. Table 2 summarises the characteristics of recruited participants at baseline. Fifteen participants (5.75%) had two comorbid illnesses which were distributed as follows; 9 (3.44%) patients had comorbid epilepsy and psychosis, 3 (1.15%) patients had comorbid psychosis and substance misuse disorders, and one patient each had comorbid epilepsy and dementia (0.38%), psychosis and other significant mental health complaints (0.38%) and depression and suicide (0.38%).

**Table 2:** Baseline sociodemographic characteristics of patients. Data are presented as n (%) for categorical variables and as mean (SD), median (IQR), and range for age. Percentages were calculated using the total baseline sample (N = 261), except where data were missing.

Characteristic	Category	n (%) or summary
Age (years)	Mean (SD)	35.68 (14.15)
	Median (IQR)	33 (25–43)
	Range	18–84
Sex	Female	160 (61.30)
	Male	101 (38.70)
Marital status	Single	142 (54.41)
	Married	80 (30.65)
	Separated/divorced	22 (8.43)
	Widowed	17 (6.51)
Education level	None	80 (30.65)
	Primary	139 (53.26)
	Secondary	31 (11.88)
	Tertiary	11 (4.21)
Referral source	Community health volunteer	27 (10.42)
	Self-referral	198 (76.45)
	Traditional healer	34 (13.13)
	Missing	2 (0.77)

*(i) Changes in levels of disability*

Table 3 summarises the patient level outcomes. In the adjusted mixed-effects model, the intervention was associated with a significant reduction in disability scores (β = −5.08, 95% CI −6.19, −3.98, p < 0.001), indicating improved disability outcomes among patients exposed to the intervention.

**Table 3:** Adjusted intervention effects on patient-level outcomes. Results are from adjusted mixed-effects models with fixed effects for treatment and study period and random intercepts for facility and participant.

Outcome	β	95% CI	p-value
Disability	−5.08	−6.19 to −3.98	<0.001
Quality of life – Physical	6.26	4.17 to 8.35	<0.001
Quality of life – Psychological	4.56	2.35 to 6.77	<0.001
Quality of life – Social	2.44	−0.96 to 5.84	0.160
Quality of life – Environment	8.17	6.18 to 10.17	<0.001
Symptom improvement since previous consultation	−0.82	−0.94 to −0.71	<0.001
Treatment efficacy	−2.22	−2.66 to −1.77	<0.001
Severity of illness at consultation	−0.64	−0.79 to −0.49	<0.001

Abbreviation: CI, confidence interval.

*(ii) Changes in quality of life*

The intervention was also associated with significant improvements in quality of life in three of the four domains assessed. Compared with the control condition, intervention exposure was associated with higher physical quality-of-life scores (β = 6.26, 95% CI 4.17, 8.35, p < 0.001), higher psychological scores (β = 4.56, 95% CI 2.35, 6.77, p < 0.001), and higher environmental scores (β = 8.17, 95% CI 6.18, 10.17, p < 0.001). Although social quality-of-life scores were higher in the intervention group, this difference did not reach statistical significance (β = 2.44, 95% CI = −0.96, 5.84, p = 0.16*).*

*(iii) Changes in severity of illness*

For clinical severity outcomes, intervention exposure was associated with greater symptom improvement since the previous consultation (β = −0.82, 95% CI −0.94, −0.71, p < 0.001), greater treatment efficacy (β = −2.22, 95% CI −2.66, −1.77, p < 0.001), and lower severity of illness at consultation (β = −0.64, 95% CI −0.79, −0.49, p < 0.001). Overall, the patient-level analyses showed that intervention exposure was associated with lower disability, improvement in three quality-of-life domains, and better clinical severity outcomes.

## Discussion

A comprehensive 10-day training on the use of the mhGAP-IG, coupled with quarterly face-to-face supportive supervision meetings and need-based supportive supervision improved clinical detection of priority MNS in primary care facilities in rural coastal Kenya. It also reduced trained primary healthcare workers’ levels of stigma against people with MNS and improved patient outcomes. Review of literature did not identify any other study that has conducted large-scale implementation of all the modules of the mhGAP-IG in real life clinical settings, using a stepped-wedge design. While similar findings from other studies suggest potential applicability in comparable contexts, these studies implemented singular modules of the guidelines in different settings and none used a stepped wedge design [15–18]. Therefore, the generalizability of our findings beyond Kilifi County should be interpreted with caution, and replication in other low-resource settings is required.

Primary healthcare workers’ improvements in clinical consultations for common mental disorders post-training are consistent with other studies. Importantly, our study found significant improvements in the proportion of clinical consultations for neurological disorders (epilepsy and dementia) for which there is a large treatment gap in low resource settings. Our findings on epilepsy have been observed in other low-resource settings in sub-Saharan Africa [16] but our study included training on non-convulsive epilepsies which may have improved the epilepsy diagnostic gap in this setting. There is emerging evidence on the efficacy of the mhGAP-IG in improving dementia care in primary care facilities in low-resource settings [19] and our study provides evidence of its application in real-life clinical settings. Low- and middle-income countries are home to approximately two-thirds of people with dementia [20] and this is expected to rise to more than 70% by 2050 hence the need to accelerate the adoption of task-sharing approaches for dementia care in primary care facilities.

The non-significant findings for suicidality and psychosis should be interpreted in the context of the study’s power calculations which were designed around the primary composite outcome rather than disorder specific detection rates. For suicidality, the extremely low baseline detection rates reflects well documented structural barriers to reporting in this setting such as (until recently), the criminalization of suicide in Kenya coupled with sociocultural issues that perpetuate stigma against survivors of suicidal behaviour [21]. For psychosis, the non-significant increase likely reflects the entrenched referral culture in this setting whereby patients with psychotic disorders are directed to tertiary facilities without documentation at the primary care level. The wide confidence intervals for both disorders reflect statistical uncertainty rather than absence of effect, and both findings warrant further investigation in studies adequately powered for disorder-specific analysis.

Stigma has been identified as a barrier to successful implementation of the mhGAP-IG [22–24]. Post-training, we found a reduction in PHW’s levels of public stigma against people with MNS which may have contributed to increased assessment and treatment by tackling work competency threats.

The implementation design had several strengths. Prior to implementing this study, we contextualized, and pilot-tested the mhGAP-IG with important modification to the original training manual. Firstly, unlike the recommended 5-day training, the intervention was delivered over a 10-day period and majority of this additional time was allocated to role plays and tackling case scenarios which may have boosted the primary healthcare workers (PHW) confidence in assessing MNS cases [7]. One study in Ethiopia conducted a ten-day training for the depression module, but in that study, trainees spent five days shadowing a specialist [25]. Additionally, the epilepsy module was expanded to include non-convulsive epilepsy which is poorly recognised in primary care which may have improved the PHW diagnostic precision. We implemented all modules of the guidelines except the child and adolescent mental and behaviour disorders module which improved primary healthcare workers likelihood of assessing and managing comorbid conditions resulting in better patient outcomes. Indeed 6% of the patients who were randomly recruited for patient level follow up were diagnosed with comorbid MNS.

The WHO mhGAP team members oversaw the training process which provided external validity to the fidelity of training. Secondly, specialists who participated in the contextualization and piloting were included in the study as supervisors. We also allowed unstructured telephone-based contact between them, and the trainees. This increased availability of referral pathways and minimised potential risks of misdiagnosis or poor patient management. Our supervision plan was semi-structured; we only facilitated the quarterly face-to-face meetings to review complex cases, but we did not monitor or evaluate the frequency, duration, content or outcomes of the teleconsultations between the trained healthcare workers and the specialists. It is therefore possible that in some cases the supervisors conducted full assessments and provided treatment plans via telephone. This may be viewed as a reflection of a practical approach to supportive supervision rather than as a confounder to the primary outcome. It also provides opportunities for exploring the potential of specialist teleconsultations at primary care facilities, a concept which is already being applied across many low-resource settings, including other parts of Kenya [26]

The sustainability of training and supervision is an important consideration for scale-up. While the intervention demonstrated effectiveness within the study period, it is unclear whether similar outcomes could be maintained without ongoing supervision and refresher support. Continued investment in training, supervision, and integration into routine health systems will likely be necessary to sustain gains in detection and patient outcomes over time. Emerging efforts within the county health system to support mhGAP-IG implementation are encouraging, but long-term sustainability remains to be evaluated.

This study had limitations. We recruited 127 instead 128 primary healthcare facilities. This reduced our average cluster size from the anticipated 8 to 7.4. However, this number was still sufficiently powered to detect significant differences in the proportions of clinical consultations for MNS diagnoses. The sample size calculation assumed an intracluster correlation coefficient (ICC) of 0.05 based on empirical estimates. However, sensitivity analyses using alternative ICC values were not conducted, and the precision of the estimates may be affected if the true ICC differs from this assumption. We provided no quota restrictions to the types of disorders that could be recruited to patient level studies therefore we cannot provide disorder specific analyses of mhGAP-IG effectiveness in improving functional outcomes for disorders such as dementia, substance misuse and suicidality which were underrepresented in the data. We relied on routine records to collect primary outcome data. Previous studies have identified poor record keeping as a health system challenge in many low-resource setting [27] therefore it is possible that we may have underestimated the primary outcome. We cannot exclude the possibility that awareness of training status among healthcare workers may have introduced a Hawthorne effect, whereby improvements in MNS detection partly reflected heightened clinical vigilance rather than the training intervention alone. This represents a potential source of performance bias inherent in pragmatic implementation trials where blinding of providers is not feasible. In this paper, we have not presented results of process evaluation, cost effectiveness or cost benefit analysis, therefore it is not possible to make finance-related policy recommendations, or information about barriers and facilitators to successful implementation of the guidelines. These are key determinants of translation of research findings to policy and practise. However, these analyses are underway and will be presented as a separate paper. We did not conduct sensitivity analyses to measure the impact of the COVID-19 pandemic on diagnoses as this was not specified *a priori*. Although the stepped-wedge design accounts for secular trends through time-fixed effects in the statistical model, we cannot fully exclude residual effects of the COVID-19 pandemic on health seeking behaviour, facility attendance and healthcare worker capacity, particularly during the early intervention period. Severity scales were clinician-rated which may have introduced reporting bias but there were no reports of adverse events such as misdiagnosis and poor patient management suggesting that overall treatment was efficacious in improving patient functionality.

In conclusion, this stepped wedge cluster randomized trial provides context-specific evidence from rural coastal Kenya on the feasibility and potential effectiveness of large scale mhGAP-IG implementation in lowering the treatment gap for common MNS disorders in primary care. Importantly, we demonstrated the utility of training primary healthcare workers on all the modules of the mhGAP-IG. Our finding of a reduction in the reporting of other significant mental health complaints accompanied by an increase in diagnoses for epilepsy, depression, dementia and substance misuse disorders and significant improvement in patient outcomes indicated improved quality of care. There are several reports of successful integration of care for mental disorders in primary care in low-resource settings [6] but data on neurological disorders (epilepsy and dementia) are few; our study provides these useful data that can inform future studies. While the study provides a promising foundation for considering scale-up of mhGAP-IG training within similar primary care systems, caution is warranted in extrapolating these findings beyond settings with comparable health system configurations, workforce cadres and cultural contexts. Replication across diverse low-resource settings remains necessary before broader policy recommendations for scale up can be made.

## Supporting information

S1 Table Baseline and post-intervention scores for the mhGAP-IG multiple choice questions. (DOCX)

S2 Table Primary healthcare workers’ change in levels of knowledge as measured by the Mental Awareness Knowledge Schedule (MAKS) (n = 172). (DOCX)

S3 Table Primary healthcare workers’ change in intended behavior as measured by the Reported and Intended Behaviors Scale (RIBS) (n = 172). (DOCX)

S4 Table Primary healthcare workers’ changes in attitudes as measured by the Community Attitudes Towards the Mentally Ill Scale (CAMI) (n = 172). (DOCX)
