# Supplementary material for: Reducing the diagnostic and treatment gap for priority mental, neurological and substance misuse disorders in primary care in rural Kenya: Results of a stepped wedge cluster randomized trial from the EPINA study
Source: PLoS One. 2026 Aug 17;21(8):e0352643. doi: 10.1371/journal.pone.0352643 (PMC13480642; doi:10.1371/journal.pone.0352643)
Supplement: Supplementary file 1 [file pone.0352643.s001.docx]

## Supplementary table S1. Baseline and post intervention scores for the mhGAP-IG multiple choice questions

| **Module** | **Baseline median score** | **Postintervention score (Interquartile range** | **P value for test of equality of medians** |
| --- | --- | --- | --- |
| ECP | 66.7 (53.3,80) | 86.7 (73.3,93.3) | 0.000 |
| Depression | 80 (70,90) | 90 (90,100) | 0.000 |
| Psychosis | 77.8 (66.7,88.9) | 94.4 (88.9,100) | 0.000 |
| Epilepsy | 85.7 (71.4,92.9) | 92.6 (85.7, 100) | 0.000 |
| Dementia | 90.9 (72.7,90.9) | 100 (100,100) | 0.000 |
| Suicide | 75 (62.5,87.5) | 100 (87.5,100) | 0.000 |
| Substance use disorders | 83.3 (66.7,91.7) | 91.7 (83.3,100) | 0.000 |
| Other significant mental health complaints | 62.5 (37.5,75) | 93.8 (87.5,100) | 0.000 |
