# Supplementary material for: Reducing the diagnostic and treatment gap for priority mental, neurological and substance misuse disorders in primary care in rural Kenya: Results of a stepped wedge cluster randomized trial from the EPINA study
Source: PLoS One. 2026 Aug 17;21(8):e0352643. doi: 10.1371/journal.pone.0352643 (PMC13480642; doi:10.1371/journal.pone.0352643)
Supplement: Supplementary file 2 [file pone.0352643.s002.docx]

## Supplementary table S2. Primary healthcare workers change in levels of knowledge as measured by the Mental Awareness Knowledge Schedule (MAKS), n=172

|  | **Univariable analysis** | |  | | **Multivariable analysis** | |  | |
| --- | --- | --- | --- | --- | --- | --- | --- | --- |
| **Variable** | **General mental health related knowledge** | | **Knowledge about specific mental illnesses** | | **General mental health related knowledge** | | **Knowledge about specific mental illnesses** | |
|  | β coefficient (95%CI) | P>z | β coefficient (95%CI) | P value | β coefficient (95%CI) | P value | β coefficient (95%CI) | P value |
| **Stage** | | | | | | | | |
| Baseline (ref) | - | - | - | - | - | - | - | - |
| Time 2 | 0.72(0.51,0.93) | 0.00 | 0.17(-0.06,0.39) | 0.15 | 0.74 (0.52,0.96) | 0.00 | 0.18(-0.05,0.42) | 0.12 |
| **Age** | 0.01(-0.01,0.03) | 0.41 | 0.01(-0.01,0.03) | 0.37 | 0.01(-0.01,0.03) | 0.32 | 0.01(-0.01,0.03) | 0.41 |
| **Sex** | | | | | | | | |
| **Fe**male (ref) | - | - | - | - | - | - | - | - |
| Male | -0.07(-0.31,0.17) | 0.57 | 0.08(-0.16,0.32) | 0.50 | -0.02(-0.25,0.21) | 0.84 | 0.08(-0.17,0.33) | 0.52 |
| **Education** | 0.11(-0.15,0.37) | 0.40 | -0.17(-0.43,0.08) | 0.19 | 0.14(-0.12,0.39) | 0.29 | -0.12(-0.39,0.15) | 0.39 |
| **Designation** | | | | | | | | |
| 1 (ref) | - | - | - | - | - | - | - | - |
| 2 | -0.05(-0.31,0.21) | 0.71 | -0.14(-0.41,0.12) | 0.29 | -0.07(-0.32,0.18) | 0.58 | -0.13(-0.39,0.14) | 0.34 |
