# Supplementary material for: Reducing the diagnostic and treatment gap for priority mental, neurological and substance misuse disorders in primary care in rural Kenya: Results of a stepped wedge cluster randomized trial from the EPINA study
Source: PLoS One. 2026 Aug 17;21(8):e0352643. doi: 10.1371/journal.pone.0352643 (PMC13480642; doi:10.1371/journal.pone.0352643)
Supplement: Supplementary file 3 [file pone.0352643.s003.docx]

## Supplementary table S3. Primary healthcare workers change in intended behaviour as measured by the Reported and Intended Behaviours Scale (RIBS) n=172

| **Variable** | **Univariable analysis** | | **Multivariable analysis** | |
| --- | --- | --- | --- | --- |
|  | β coefficient (95%CI) | P value | β coefficient (95%CI) | P value |
| **Stage** |  |  |  |  |
| Baseline (ref) | - | - | - | - |
| Time 2 | 0.72(0.52,0.92) | 0.00 | 0.68(0.48,0.88) | 0.00 |
| Age | 0.01(-0.01,0.03) | 0.26 | 0.01(-0.00,0.03) | 0.13 |
| **Sex** |  |  |  |  |
| Female (ref) | - | - | - | - |
| Male | -0.09(-0.31,0.13) | 0.41 | -0.02(-0.24,0.19) | 0.83 |
| **Education** | 0.24(-0.00,0.48) | 0.05 | 0.26(0.02,0.49) | 0.03 |
| **Designation** |  |  |  |  |
| Nurse | - | - | - | - |
| Clinical Officer | 0.06(-0.18,0.30) | 0.63 | 0.02(-0.21,0.25) | 0.85 |
