# Supplementary material for: Reducing the diagnostic and treatment gap for priority mental, neurological and substance misuse disorders in primary care in rural Kenya: Results of a stepped wedge cluster randomized trial from the EPINA study
Source: PLoS One. 2026 Aug 17;21(8):e0352643. doi: 10.1371/journal.pone.0352643 (PMC13480642; doi:10.1371/journal.pone.0352643)
Supplement: Supplementary file 4 [file pone.0352643.s004.docx]

## Supplementary table S4. Primary healthcare workers’ changes in attitude as measured by the Community Attitudes Towards the Mentally Ill Scale (n=172)

|  | **Univariable analysis** | | | | | | **Multivariable analysis** | | | | | |
| --- | --- | --- | --- | --- | --- | --- | --- | --- | --- | --- | --- | --- |
| **Variable** | **Liberal attitude** | | **Tolerant attitude** | | **Sympathetic attitude** | | **Liberal attitude** | | **Tolerant attitude** | | **Sympathetic attitude** | |
|  | β coefficient (95%CI) | P value | β coefficient (95%CI) | P value | β coefficient (95%CI) | P value | β coefficient (95%CI) | P value | β coefficient (95%CI) | P value | β coefficient (95%CI) | P value |
| **Stage** | | | | | | | | | | | | |
| Baseline (ref) | - | - | - | - | - | - | - | - | - | - | - | - |
| Time 2 | 0.65(0.44,0.86) | 0.00 | 0.49(0.27,0.71) | 0.00 | 0.53(0.32,0.75) | 0.00 | 0.68(0.46,0.89) | 0.00 | 0.51(0.28,0.73) | 0.00 | 0.52(0.30,0.75) | 0.00 |
| **Age** | -0.01(-0.03,0.01) | 0.50 | 0.01(-0.01,0.03) | 0.52 | 0.02(-0.00,0.03) | 0.12 | -0.01(-0.03,0.01) | 0.34 | 0.00(-0.02,0.02) | 0.69 | 0.02(-0.00,0.04) | 0.08 |
| **Sex** | | | | | | | | | | | | |
| **Fe**male (ref) | - | - | - | - | - | - | - | - | - | - | - | - |
| Male | 0.11(-0.12,0.34) | 0.35 | -0.09(-0.33,0.15) | 0.47 | -0.09(-0.33,0.15) | 0.45 | 0.04(-0.18,0.27) | 0.71 | -0.12(-0.36,0.12) | 0.32 | -0.04(-0.28,0.20) | 0.74 |
| **Education** | -0.14(-0.39,0.11) | 0.28 | -0.17(-0.43,0.10) | 0.22 | 0.14(-0.11,0.40) | 0.27 | -0.20(-0.45,0.05) | 0.11 | -0.20(-0.47,0.07) | 0.15 | 0.15(-0.11,0.41) | **0.25** |
| **Designation** | | | | | | | | | | | | |
| 1 (ref) | - | - | - | - | - | - | - | - | - | - | - | - |
| 2 | 0.31(0.05,0.56) | 0.02 | -0.05(-0.31,0.40) | 0.71 | 0.17(-0.09,0.43) | 0.20 | 0.34(0.10,0.58) | 0.01 | -0.02(-0.27,0.24) | 0.91 | 0.15(-0.11,0.40) | 0.26 |
